# Therapeutic approaches in chronic fatigue syndrome

**DOI:** 10.1192/j.eurpsy.2021.1163

**Published:** 2021-08-13

**Authors:** O. Vasiliu

**Affiliations:** Psychiatry, University Emergency Central Military Hospital Dr. Carol Davila, Bucharest, Romania

**Keywords:** chronic fatigue syndrome, major depressive disorder, antidepressants, psychotherapy

## Abstract

**Introduction:**

Chronic fatigue syndrome (CFS) is a complex condition, with an insufficiently known pathophysiology, that raises multiple challenges to the treating physicians. Due to its yet mostly unknown underlying mechanisms, there is no consensus treatment recommendation for CFS. The risk to associate major depression, anxiety disorders or substance use disorders is frequently reported, and this co-morbidity further complicates the evolution of CFS.

**Objectives:**

To search the existing literature for pharmacological and psychotherapeutic recommendations in CFS.

**Methods:**

A literature search was performed using the main electronic databases using the paradigm „chronic fatigue syndrome” AND „psychopharmacological treatment” OR „psychotherapy”. All papers published between January 2000 and August 2020 were included in the primary analysis.

**Results:**

Anti-inflamatory drugs (corticosteroids and non-steroidal drugs), antidepressants, moodstabilizers, anxiolytics, immuno-modulatory drugs, and antivirals have been investigated for CFS, but the trials had low-quality designs, used various definition of CFS, and different criteria for monitoring the efficacy of treatment. Cognitive behavioral therapy (CBT) may be promising for decreasing the fatigue severity, but larger trials are needed. Graded exercise therapy (GET) also may be of some use for improving patients ability to engage in activities, but caution should be in order because of the risk of over-exercising that may exacerbate the core CFS symptoms.

**Conclusions:**

Larger trials are needed in order to validate pharmacological and psychotherapeutic recommendations for CFS. No drug may be considered first line treatment for this indication, while CBT and GET may be useful, although they do not address all the central symptoms of CFS.

